# Bedaquiline and levofloxacin replacing rifampicin for the treatment of TB

**DOI:** 10.5588/ijtldopen.25.0263

**Published:** 2025-09-10

**Authors:** C.-Y. Chiang, K-J. Bai, M.-C. Yu

**Affiliations:** ^1^ Division of Pulmonary Medicine, Department of Internal Medicine, Wan Fang Hospital, Taipei Medical University, Taipei, Taiwan;; ^2^ Department of Internal Medicine, School of Medicine, College of Medicine, Taipei Medical University, Taipei, Taiwan;; ^3^ International Union against Tuberculosis and Lung Disease, Paris, France;; ^4^ School of Respiratory Therapy, College of Medicine, Taipei Medical University, Taipei, Taiwan.

**Keywords:** tuberculosis, BDQ, MDR-TB, drug susceptibility testing

Dear Editor,

Rifampicin-resistant TB (RR-TB) and multidrug-resistant TB (MDR-TB) are different in terms of their susceptibility to isoniazid (I or INH), which is resistant in MDR-TB but susceptible in RR-TB. However, the WHO recommends managing RR-TB as MDR-TB,^1,2^ in part because susceptibility to INH may be unknown and because regimens for RR-TB have not been specified. In 2022, WHO recommended the BPaLM regimen consisting of bedaquiline (B or BDQ), linezolid (L), pretomanid (Pa) and moxifloxacin (M) for the management of MDR/RR-TB.^2,3^ Recently, WHO recommended a 6-month regimen (BDLLfxC) composed of bedaquiline, delamanid (D), linezolid, levofloxacin (Lfx), and clofazimine (C) in MDR/RR-TB patients with or without fluoroquinolone resistance, in which clofazimine may be discontinued if fluoroquinolone is susceptible.^4,5^ WHO also recommended three 9-month regimens, namely BLMZ (bedaquiline, linezolid, moxifloxacin, pyrazinamide), BLLfxCZ (bedaquiline, linezolid, levofloxacin, clofazimine, pyrazinamide), and BDLLfxZ (bedaquiline, delamanid, linezolid, levofloxacin, pyrazinamide) for the management of MDR/RR-TB patients with confirmed drug susceptibility to fluoroquinolones.^4,5^ All these recommended regimens contain linezolid which is associated with frequent adverse reactions, including bone marrow suppression and neurotoxicity.^6^ INH has potent early bactericidal activity.^7^ The approach to manage RR-TB as MDR-TB fails to take the utility of INH into account. A study analyzing data of the Global Project on Anti-Tuberculosis Drug Resistance Surveillance reported that among rifampicin-resistant isolates from new TB patients, the proportion of isolates that was susceptible to INH varied between settings, ranging from 14% in settings with relatively high MDR-TB prevalence to 43% in settings with relatively low MDR-TB prevalence.^8^ WHO estimated that among 465,000 incident cases of MDR/RR-TB in 2020 globally, 22% were RR-TB.^9^ Proper use of INH for the treatment of RR-TB may avoid the use of linezolid.

Pulmonary TB (PTB) patients who experienced intolerance to rifampicin due to adverse reactions may be treated with rifabutin.^10^ Patients who do not tolerate rifabutin need to be treated with rifamycin-sparing regimens, such as INH, ethambutol and pyrazinamide with or without a fluoroquinolone for 12 months.^11^ Based on the results of STREAM stage 2 trial,^12,13^ we hypothesize that the combination of BDQ and a fluoroquinolone may be as efficacious as rifampicin, and that BDQ combined with a fluoroquinolone may replace rifampicin and ethambutol in the standard 6-month rifampicin-based regimen for the treatment of PTB. Bases on this hypothesis, we designed a novel regimen consisted of INH, BDQ and a fluoroquinolone throughout for 6 months, supplemented by pyrazinamide for the initial 2 months. The duration of treatment may be prolonged to 9 months as needed. The proposed study regimen obtained approval of Joint Institutional Review Board of Taipei Medical University (N202405123), with informed consent of the two TB patients.

## CASE 1

The first patient was a 55 year-old female diagnosed with smear-negative culture-positive PTB in November 2022 at Wan Fang Hospital, Taipei Medical University. Drug susceptibility testing showed susceptibility to INH, rifampicin, ethambutol, pyrazinamide and streptomycin. She was treated with a standard regimen consisted of INH, rifampicin, ethambutol and pyrazinamide. On day 11, she had fever and general discomfort, and elevation of aspartate aminotransferase/alanine aminotransferase from 18/15 U/L at baseline to 53/84 U/L. The anti-TB regimen was discontinued temporarily. On day 18, INH 50 mg daily was re-introduced, with gradual escalation to 300 mg daily. On day 22, levofloxacin 500 mg daily was added, and increased to 750 mg daily later. On day 30, rifampicin 150 mg was re-challenged, resulting in chills, fever, nausea and vomiting in a few hours. Therefore, rifampicin was suspended, and she continued to take levofloxacin and INH. On day 33, rifabutin 75mg daily was tried, with gradual escalation to 300 mg daily. On day 44, she had fever and general discomfort resulting in suspension of all anti-TB drugs. On day 47, INH and levofloxacin were re-introduced. On day 48, ethambutol was re-introduced. On day 53, pyrazinamide was re-introduced. On day 60, BDQ was introduced. On day 71, ethambutol was discontinued. Pyrazinamide was applied for two months. The patient knew that she was the first patient treated with this novel regimen and whether treatment duration should be 6 or 9 months was uncertain. Under close monitoring, she had a good response to treatment and sputum culture conversion took place at month one. A follow-up chest computed tomography (CT) scan at month 5 of BDQ treatment revealed almost complete regression of lung parenchyma lesions as compared with the initial chest CT. However, she suffered from soreness of bilateral arms, shoulders and proximal interphalangeal joints of bilateral hands. She also had discomfort of bilateral soles of her foot, which made it painful to stand and walk. Because of the soreness and painful sensation, she asked whether treatment could be discontinued as early as possible. On day 212, the case was presented at an expert committee meeting organized by Taiwan Centers for Disease Control to determine the duration of treatment. Of the 13 experts who participated, 10 recommended that treatment be discontinued after 6-month treatment with BDQ; 3 recommended that BDQ be discontinued after 6 months, but INH and levofloxacin could be continued for a total duration of 9 months. Aligned with the principles of shared decision-making, she was informed of the results of discussion. She understood that the painful sensation was likely due to levofloxacin and was not sure about whether to continue the suffering of soreness or to take the small risk of TB relapse with a 6-month treatment. After thorough consideration, she decided to continue treatment with INH and levofloxacin for a total period of 9 months. However, on day 240, she asked to discontinue TB treatment. She returned for 16-month post treatment follow-up in December 2024 and there was no evidence of recurrent TB.

## CASE 2

The second patient was an 82 year-old man who had smear-positive miliary TB that was culture positive for *M tuberculosis.* Drug susceptibility testing showed susceptible to INH, rifampicin, ethambutol, pyrazinamide, and streptomycin. He was treated with a rifampicin-containing standard regimen initially but had drug-induced thrombocytopenia and leukocytopenia. Anti-TB treatment was suspended temporarily, followed by sequential re-introduction of INH, pyrazinamide ethambutol, levofloxacin and BDQ. Ethambutol was stopped following the implementation of the proposed regimen, while pyrazinamide was used for the initial 2 months. A follow-up chest CT scan at month 5 of BDQ treatment revealed almost complete regression of lung parenchyma lesions.The case was also presented at an expert committee meeting. Of the 13 experts who participated, 12 recommended that treatment may be discontinued after 6-month of BDQ and one made no comment. The patient and his family were informed of the recommendations from the committee. Two options were proposed for their consideration: either to stop treatment after completion of 6-month BDQ or to extend treatment with INH and levofloxacin till completion of 9-month levofloxacin. They opted to stop anti-TB treatment after completion of 6-month BDQ. He returned for 16-month post treatment follow-up in February 2025 and there was no evidence of recurrent TB.

Sustained treatment success of these two cases was encouraging, indicating that it might be worthwhile to test this novel regimen in a larger study population. We have initiated a clinical trial to test this regimen in patients with RR-TB and patients unable to tolerate rifampicin in late 2024 (ClinicalTrials.gov Identifier: NCT06526039).
